# Phenolic Profile of Dark-Grown and Photoperiod-Exposed *Hypericum perforatum* L. Hairy Root Cultures

**DOI:** 10.1155/2013/602752

**Published:** 2013-12-26

**Authors:** Oliver Tusevski, Jasmina Petreska Stanoeva, Marina Stefova, Sonja Gadzovska Simic

**Affiliations:** ^1^Department of Plant Physiology, Institute of Biology, Faculty of Natural Sciences and Mathematics, “Ss. Cyril and Methodius” University, P.O. Box 162, 1000 Skopje, Macedonia; ^2^Department of Analytical Chemistry, Institute of Chemistry, Faculty of Natural Sciences and Mathematics, “Ss. Cyril and Methodius” University, P.O. Box 162, 1000 Skopje, Macedonia

## Abstract

*Hypericum perforatum* L. is a medicinal plant considered as an important natural source of secondary metabolites with a wide range of pharmacological attributes. Hairy roots (HR) were induced from root segments of *in vitro* grown seedlings from *H. perforatum* after cocultivation with *Agrobacterium rhizogenes* A4. Investigations have been made to study the production of phenolic compounds in dark-grown (HR1) and photoperiod-exposed (HR2) cultures. The chromatographic analysis of phenolic acids, flavonols, flavan-3-ols, and xanthones revealed marked differences between HR1 and HR2 cultures. The production of quinic acid, kaempferol, and seven identified xanthones was increased in HR2. Moreover, HR2 showed a capability for *de novo* biosynthesis of two phenolic acids (3-p-coumaroylquinic acid and 3-feruloylquinic acid), three flavonol glycosides (kaempferol hexoside, hyperoside, and quercetin acetylglycoside), and five xanthones (tetrahydroxy-one-methoxyxanthone, 1,3,5-trihydroxy-6-methoxyxanthone, 1,3,5,6-tetrahydroxy-2-prenylxanthone, paxanthone, and banaxanthone E). On the other side, HR1 cultures were better producers of flavan-3-ols (catechin, epicatechin, and proanthocyanidin dimers) than HR2. This is the first comparative study on phenolic profile of *H. perforatum* HR cultures grown under dark and photoperiod conditions.

## 1. Introduction

*Hypericum perforatum* L. (St. John's wort) is a traditional medicinal plant with a complex mixture of secondary metabolites. Phenolic compounds as naphthodianthrones, acylphloroglucinols, flavonoids, and xanthones are the main bioactive metabolites commonly described for this plant [1]. In phytomedicine, *Hypericum* extracts are responsible for a plethora of pharmacological activities including antidepressant, antiviral, antioxidant, anti-inflammatory, and antimicrobial properties [2]. To meet the increasing demand for plants utilized in the pharmaceutical industry, much of the recent research has focussed on the development of new *in vitro* culture techniques as a useful alternative to improve the yield of bioactive metabolites in plant material.

*Agrobacterium rhizogenes*-mediated plant transformation represents a convenient experimental system for establishment of hairy roots (HR). Transformed root cultures represent an attractive model system for the production of high-value secondary metabolites, including pharmaceuticals and other biologically active substances of commercial importance [3]. Namely, HR cultures may synthesize higher levels of secondary metabolites or amounts comparable to those of the intact plant and offer a promising approach to the industrial exploitation of HR for production of novel metabolites [4, 5]. Until now, only *A. rhizogenes*- [6, 7] and biolistic-mediated [8] transformation methods have been applied. In recent years, it has been shown that HR are responsive to physical stimuli such as exposure to light which is known to regulate a number of plant developmental processes [9], as well as primary and secondary metabolite production [10]. These findings indicate that the exposure of HR to light leads to alternations in their biosynthetic potentials. Although several studies investigated secondary metabolite production in root cultures [11, 12], the capacity of *H*.* perforatum* HR to produce phenolic compounds has never been explored.

This study describes the phenolic profile of transformed roots (HR) from *H. perforatum* transformed with *A. rhizogenes *strain A4, grown in constant dark (HR1) or in light/dark photoperiod (HR2) conditions. Phenolic compounds in HR were analyzed using high-performance liquid chromatography (HPLC) coupled with diode array detection (DAD) and tandem mass spectrometry (MS^*n*^) with electrospray ionization (ESI). All present derivatives of phenolic acids, flavonol glycosides, flavonoid aglycones, flavan-3-ols, and xanthones were identified from corresponding UV and MS spectra and quantified by HPLC-DAD.

## 2. Material and Methods

### 2.1. Plant Material

Seeds from *H. perforatum* were collected from wild plants growing in a natural population in the Pelister National Park at about 1394 m. Voucher specimen (number 060231) of *H. perforatum* is deposited in the Herbarium at the Faculty of Natural Sciences and Mathematics, University “Ss. Cyril and Methodius,” Skopje, Macedonia. As for a previous study [13], seeds were surface sterilized and *in vitro* germinated seedlings were maintained in a growth chamber at 25 ± 1°C under a photoperiod of 16 h light, irradiance at 50 *μ*mol · m^2^ · s^−1^, and 50 to 60% relative humidity.

### 2.2. Establishment of Hairy Roots

The wild type *Agrobacterium rhizogenes* agropine strain A4 (obtained from Institut National de la Recherche Agronomique-INRA, Versailles, France) was used for *H. perforatum* transformation experiments [14]. Transformation protocol was performed according to Di Guardo et al. [6] with the modifications described in our previous study [15]. Briefly, the HR cultures were induced by *A. rhizogenes* A4 from root segments of one-month-old *in vitro* germinated seedlings from *H. perforatum*. Transgenic status of the HR was confirmed by PCR analysis using *rol*B specific primers [15]. Transformed root cultures were maintained by subculturing at one-month intervals on MS/B_5_ hormone-free medium. The subculture was carried out at 25 ± 1°C in the dark (HR1) and under photoperiod (HR2) of 16 h light (50 *μ*mol · m^2^ · s^−1^). One-month-old HR1 and HR2 cultures were harvested (1 g) and then frozen in liquid nitrogen or lyophilized and stored at −80°C, until analysis.

### 2.3. HPLC/DAD/ESI-MS^*n*^ Analysis

Phenolic compounds extraction from freeze-dried lyophilized and powdered root cultures was performed as previously reported by Tusevski et al. [15]. The HPLC system was equipped with an Agilent 1100 series diode array and mass detector in series (Agilent Technologies, Waldbronn, Germany). It consisted of a G1312A binary pump, a G1313A autosampler, a G1322A degasser, and a G1315B photodiode array detector, controlled by ChemStation software (Agilent, v.08.03). Chromatographic separations were carried out on 150 mm × 4.6 mm, 5 *μ*m XDB-C18 Eclipse column (Agilent, USA). The mobile phase consisted of two solvents: water-formic acid (A; 99 : 1, v/v) and methanol (B) in the following gradient program: 90% A and 10% B (0–20 min), 80% A and 20% B (20–30 min), 65% A and 35% B (30–50 min), 50% A and 50% B (50–70 min), and 20% A and 80% B (70–80 min) and continued with 100% B for a further 10 min. Each run was followed by an equilibration period of 10 min. The flow rate was 0.4 mL/min and the injection volume was 10 *μ*L. All separations were performed at 38°C. Formic acid (HCOOH) and methanol (CH_3_OH) were HPLC grade solvents (Sigma-Aldrich, Germany). The HPLC-water was purified by a PURELAB Option-Q system (Elga LabWater, UK). The commercial standards chlorogenic acid, rutin, quercetin, kaempferol, catechin, epicatechin, and xanthone (Sigma-Aldrich, Germany) were used as reference compounds. The reference compounds were dissolved in 80% methanol in water. The concentration of the stock standard solutions was 1 mg · mL^−1^ and they were stored at −20°C. Spectral data from all peaks were accumulated in the range of 190–600 nm, and chromatograms were recorded at 260 nm for xanthones, at 280 nm for flavan-3-ols, at 330 nm for phenolic acids, and at 350 nm for flavonols. Peak areas were used for quantification at wavelengths where each group of phenolic compounds exhibited an absorption maximum. The HPLC system was connected to the Agilent G2445A ion-trap mass spectrometer equipped with electrospray ionization (ESI) system and controlled by LCMSD software (Agilent, v.6.1.). Nitrogen was used as nebulizing gas at a pressure-level of 65 psi and the flow was adjusted to 12 L · min⁡^−1^. Both the heated capillary and the voltage were maintained at 350°C and 4 kV, respectively. MS data were acquired in the negative ionization mode. The full scan mass covered the mass range from *m/z* 100 to 1200. Collision-induced fragmentation experiments were performed in the ion trap using helium as a collision gas, with voltage ramping cycle from 0.3 up to 2 V. Maximum accumulation time of the ion trap and the number of MS repetitions to obtain the MS average spectra were set at 300 ms and 3, respectively. Identification of the component peaks was performed by the UV/Vis, MS, and MS^2^ spectra and retention times of the abovementioned available standards.

### 2.4. Statistical Analysis

The experiments were independently repeated twice under the same conditions and all analyses were performed in triplicate. Secondary metabolite contents were expressed as mg · 100 g^−1^ dry weight (DW). Standard deviation of mean value was shown as ± S.D. The statistical analyses including calculations of means and standard deviations were performed applying Excel (Microsoft Office, 2007).

## 3. Results and Discussion

### 3.1. Establishment of Hairy Roots

As previously reported [15], *H. perforatum* HR were initiated by inoculation of root explants with *A. rhizogenes* A4. On the basis of culture conditions, selected dark-grown (HR1) and photoperiod-exposed (HR2) cultures showed differences in the morphology. Dark-grown hairy root cultures were thinner and whitish in colour showing rapid plagiotropic growth with active branching and vigorous production of elongated lateral roots. Present results confirmed that transformed roots of *H. perforatum* had characteristic traits of HR previously described by Tepfer [16]. In contrast, HR2 cultures began to turn pale green after 7 days of culture and continued to acquire green coloration during the course of subsequent growth period. Moreover, HR2 appeared intense greenish-brown after one month of culture. It was seen that the growth of HR was generally most vigorous between the 3rd and 4th weeks of the cultivation period (1 month), but their growth declined after the 5th week due to the nutrient depletion. For HPLC analysis, one-month-old HR cultures were further evaluated.

### 3.2. HPLC/DAD/ESI-MS^*n*^ Analysis

**Figure 1:**
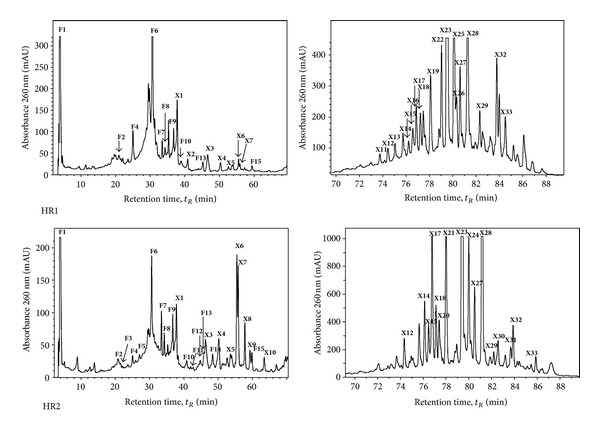
Chromatograms of *Hypericum perforatum* dark-grown (HR1) and photoperiod-exposed (HR2) hairy root culture extracts monitored at 260 nm for detection of phenolic compounds. Compound symbols correspond to those indicated in Tables 1 and 2.

The HPLC/DAD/ESI-MS^*n*^ technique was used to analyse the phenolic profile of *H. perforatum* HR1 and HR2 cultures. Four groups of phenolic compounds such as phenolic acids, flavonols, flavan-3-ols, and xanthones were recorded in HR cultures (Tables 1 and 2). The identification of phenolic compounds (Tables 1 and 2, Figure 1) was based on the typical UV/Vis spectral data and LC/MS in the negative ionization mode [M–H]^−^ with the subsequent MS^2^, MS^3^, and MS^4^ analysis for further identification with reference to similar data previously reported [15, 17–26]. The HPLC analysis of phenolic compounds revealed marked differences between HR1 and HR2 cultures (Tables 1 and 2, Figure 1).

#### 3.2.1. Phenolic Acids

Compound **F1** occurred at retention time of 3.9 min and exhibited a [M–H]^−^ ion at *m/z* 191 (Table 1, Figure 1). Its MS^2^ fragmentation produced a [M–H–CO–2H_2_O]^−^ ion at *m/z* 127 as a base peak. A [M–H–H_2_O]^−^ ion at *m/z* 173 was also observed. Compound **F1** was identified as quinic acid, taking into account its MS^*n*^ fragmentation pattern and the literature data [17]. Quinic acid (**F1**) was the only detectable phenolic acid in both HR cultures. A 1.4-fold increase of quinic acid was observed in HR2 compared to HR1 cultures.

Two peaks, 3-*p*-coumaroylquinic acid (**F3)** and 3-feruloylquinic acid (**F5)**, were detected only in HR2 cultures with identical UV spectra characterized by absorption band at 314 nm. Compounds **F3** and **F5** were readily distinguished by their cinnamic acid-derived MS^2^ base peaks at *m/z* 163 and at *m/z* 193, respectively. Quinic acid is the most important component as a key intermediate in the biosynthesis of aromatic compounds. The condensation between quinic acid and caffeic acid leads to the formation of chlorogenic acid in the shikimic acid pathway [27]. Chlorogenic acid is an important antioxidative compound recently produced by *H. perforatum* adventitious roots cultivated in bioreactor [11], shoot cultures [28], and transgenic plantlets [29].

#### 3.2.2. Flavonols

The flavonols were observed to be qualitatively and quantitatively different in both *H. perforatum* HR cultures (Table 1, Figure 1). A major identified group of compounds belonged to flavonols according to their characteristic UV spectra of flavonols glycosylated at C3 (257, 265 sh, 355 nm). The detected compound **F8** can be identified as C-glycoside of quercetin. The deprotonated molecular ion [M–H]^−^ of compound **F8** was detected at *m/z* 421. It showed MS^2^ fragmentation characteristic of mono-C-hexosyl flavones, with losses of 90 and 120 amu [19], giving *m/z* 301 ion characteristic for quercetin. The compound **F10** had a molecular ion [M–H]^−^ at *m/z* 477. The MS^2^ spectra of this compound showed fragmentation ions at *m/z* 315 (loss of 162 amu), suggesting a presence of hexose residue. So, compound **F10** was tentatively identified as isorhamnetin O-hexoside. Compound **F13** had a molecular ion [M–H]^−^ at *m/z* 609, and its MS^2^ gave a single ion at *m/z* 301, indicating quercetin derivative with rutinose at C3 [20]. The absence of intermediate fragmentation between the deprotonated molecular ion and the aglycone ion is indicative of an interglycosidic linkage 1 → 6 [21]; therefore, this compound was identified as quercetin 3-O-rutinoside (rutin). Flavonol glycosides as **F8**, **F10**, and **F13** detected in HR2 cultures were in lower amounts compared with those in HR1.

The compound **F11** was identified as kaempferol derivative with glycosylation in position 3 according to its UV-spectra (256, 266, 350 nm). The MS and MS^2^ spectra were consistent with the presence of a hexose residue and confirm the kaempferol aglycone. Therefore, this compound was identified as kaempferol hexoside. Compound **F12** had a deprotonated molecular ion [M–H]^−^ at *m/z* 463 and its MS^2^ gave a single ion at *m/z* 301, indicating quercetin hexose derivative, most probably hyperoside (quercetin 3-O-galactoside) [30]. Compound **F14** gave deprotonated molecular ion at *m/z* 505. Its MS^2^ fragmentation produced a [M–H–42]^−^ ion at *m/z* 463 and [M–H–42–162]^−^ ion at *m/z* 301 as a base peak, indicating quercetin acetylglycoside. It is worth noting, that flavonol glycosides as **F11**, **F12**, and **F14** were synthesized only in HR2 cultures.

With regard to the class of flavonol glycosides, our results showed that both HR cultures had capability to produce quercetin and kaempferol derivatives. However, there is no available study for the potential of *H. perforatum* root cultures to produce flavonol derivatives. Several differences can be pointed out when comparing the composition of flavonol glycosides in HR cultures with those of *H. perforatum in vitro* cultures. In our previous work [31, 32], we indicated that *H. perforatum* cells, calli, and shoots demonstrate a considerable potential for producing quercetin, isoquercitrin, and quercitrin upon elicitation with jasmonic acid and salicylic acid. The LC-MS screening of twelve *H. perforatum* HR transgenic plants showed a large variability in the content of rutin, hyperoside, quercetrin, and quercetin [29]. Moreover, the abovementioned flavonol glycosides had been identified in *H. perforatum* regenerated plantlets [33] and *H. undulatum* shoot cultures [34].

The HPLC-MS analysis of flavonoid aglycones in HR cultures resulted in the identification of kaempferol (**F15)**. Its molecular ion at *m/z* 285 corresponded to that of kaempferol. The identification was made by comparing its UV and MS spectra to analytical standards and literature data [28]. A 1.6-fold increase of kaempferol was observed in HR2 compared to HR1 cultures.

#### 3.2.3. Flavan-3-ols

Flavan-3-ols (catechins) were identified as the main flavonoid fraction in HR cultures. The HPLC analysis confirmed the presence of 5 flavan-3-ols: **F2**, **F4**, **F6**, **F7**, and **F9** in both HR cultures (Table 1, Figure 1). The mass spectrum in full scan mode showed the deprotonated molecules [M–H]^−^ of catechin (**F2**) and epicatechin (**F6**) at *m/z* 289, with characteristic MS^2^ ions at *m/z* 245 and 205 and UV maximum at 280 nm. Compounds **F4**, **F7**, and **F9** had [M–H]^−^ at *m/z* 577 and main fragmentation with loss of 152 amu, characteristic fragmentation pathway by retro Diels-Alder reaction [22], and were recognized as proanthocyanidin dimers. Dark-grown HR were better producers of catechins and proanthocyanidin dimers than HR2. Literature data about the production of catechin derivatives in *in vitro* cultures of *H. perforatum* are scarce. In our previous work [35], we indicated that *H. perforatum* root cultures may be considered as a promising source of proanthocyanidin dimers. Nevertheless, catechin, epicatechin, and proanthocyanidin dimers had been previously identified in shoots and calli of *H. erectum* [36] and *H. undulatum* shoot cultures [34].

#### 3.2.4. Xanthones

Xanthones comprise the majority of the phenolic compounds detected by HPLC/DAD/ESI-MS^*n*^ (Table 2, Figure 1). They include simple oxygenated xanthones or derivatives with prenyl, pyran, or methoxy groups. Compound **X1** was putatively identified as mangiferin. The HPLC-MS/MS analysis of this compound gave a molecular ion *m/z* [M–H]^−^ of 421 and major −MS^2^ fragments at *m/z* 331 [M–H–90]^−^ and 301 [M–H–120]^−^, losses characteristics of *C*-hexosyl compounds [19]. Compounds **X4**, **X6**, **X8**,** X13**, and **X20** showed UV spectral characteristics of the 1,3,5,6 oxygenated xanthones, with band IV reduced to shoulder [37], while most of the other identified xanthones had UV spectra similar to mangiferin typical of the 1,3,6,7 oxygenation pattern with a very well-defined band IV [23, 38]. Compounds **X6** and **X7** were identified as 1,3,5,6-tetrahydroxyxanthone and 1,3,6,7-tetrahydroxyxanthone aglycones, respectively (single intense molecular ion [M–H]^−^ at *m/z* 259) [20, 25]. Compounds **X4** and **X5** gave molecular ions [M–H]^−^ at *m/z* 517. Major −MS^2^ fragments at *m/z* 365 and 257, respectively, characterized them as dimers of 1,3,5,6-tetrahydroxyxanthone and 1,3,6,7-tetrahydroxyxanthone. Compound **X10** gave a molecular ion [M–H]^−^ at *m/z* 273 and major MS^2^ fragment at *m/z* 258 [M–H–15]^−^. The MS analysis indicates the presence of one methoxy and three hydroxyl groups. These together with comparison to literature data gave its identification as 1,3,5-trihydroxy-6-methoxyxanthone [26]. Compound **X8** had the same UV spectra as compound **X11** but contained one hydroxy group more and molecular ion [M–H]^−^ at 289, base peak at *m/z* 274. We tentatively assigned this compound as tetrahydroxy-one-methoxyxanthone (**X8**). Compound **X11** was putatively identified as mangiferin-*C*-prenyl isomer. The HPLC-MS/MS analysis of this compound gave molecular ions [M–H]^−^ at *m/z* 489 and major MS^2^ fragments at *m/z* 399 [M–H–90]^−^, 369 [M–H–120]^−^ with losses characteristics of *C*-hexosyl compounds [21] and 327 as a base peak (1,3,6,7-tetrahydroxyxanthone-*C*-prenyl residue). Compounds **X12** and **X17** had UV spectra characteristic of 1,3,6,7-oxygenated xanthones and molecular ions [M–H]^−^ at 327. So, these compounds were identified as 1,3,6,7-tetrahydroxyxanthone-*C*-prenyl isomers. In the literature, it is found that in some *Hypericum* species, the *C*-prenyl moiety can be in position 2 or 8 [24]. They can be tentatively assigned as 1,3,6,7-tetrahydroxy-8-prenyl xanthone (**X12**) and 1,3,6,7-tetrahydroxy-2-prenyl xanthone (**X17**). Compounds **X13** and **X20** had same fragmentation pattern as **X12** and **X17** but different UV spectra, characteristic of 1,3,5,6-tetrahydroxyxanthone, leading to their assignment as 1,3,5,6-tetrahydroxy-*C*-prenylxanthone. These compounds were identified as 1,3,5,6-tetrahydroxy-8-prenylxanthone (**X13**) and 1,3,5,6-tetrahydroxy-2-prenylxanthone (**X20**). Compound **X14** gave molecular ion [M–H]^−^ at *m/z* 327 but showed a different fragmentation pattern in comparison with the other compounds with the same mass. In the MS^2^, a loss of a hydroxyl group [M–H_2_O]^−^ to give the base peak at *m/z* 309 is exhibited, indicating that the OH group is not linked to the xanthone aglycone but to the prenyl group. In the next MS^3^ step, after the loss of the prenyl moiety, the base peak at *m/z* 257 was detected. According to this behavior and literature data [26], it is evident that this compound is 1,3,7-trihydroxy-2-(2-hydroxy-3-methyl-3-butenyl)-xanthone. Xanthones **X16** and **X19** were identified as 1,3,7-trihydroxy-6-methoxy-8-prenyl xanthone and 1,3,6-trihydroxy-7-methoxy-8-prenyl xanthone (molecular ions [M–H]^−^ at *m/z* 341), respectively, using the obtained spectral data and comparison to previously published data [20, 24, 25]. Compound **X23** had similar fragmentation pattern as compound **X16**, indicating that compound **X23** has similar nature as compound **X16**. We can tentatively term compound **X23** as trihydroxy-1-metoxy-*C*-prenyl xanthone. The comparison to previously published data [39] for UV and MS spectra indicates that compound **X25** is *γ*-mangostin (molecular ion [M–H]^−^ at *m/z* 395). Compounds **X18** and **X22** were putatively identified as isomers of *γ*-mangostin (1,3,6,7-tetrahydroxyxanthone-*C*-bis-prenyl), since they have a similar molecular ion [M–H]^−^ of 395 but different UV spectra and retention times. Compound **X15** gave a [M–H]^−^ peak at *m/z* 325. The UV spectrum was characteristic of 1,3,5,6-tetraoxygenated xanthone. A distinct shoulder at 365 nm revealed conjugation with a pyran ring. The MS^*n*^ and UV spectra were in complete agreement with those of toxyloxanthone [25]. Compound **X21** gave a [M–H]^−^ peak at *m/z* 339, which results from methylation of toxyloxanthone giving paxanthone [23, 25, 40]. Compounds **X26**, **X28**, **X30**, and **X32** gave deprotonated molecular ions [M–H]^−^ at *m/z* 461, 463, 477, and 413, respectively. Their MS^2^ spectra were generated by the loss of a prenyl residue C_4_H_8_ (56 amu) and two prenyl residues (112 amu). So, compounds **X26**, **X28**, **X30**, and **X32 **were identified as banaxanthone D, garcinone E, banaxanthone E, and garcinone C, respectively. Several other peaks (**X2**, **X3**, **X9**, **X24**, **X27**, **X29**, **X31**, and **X33**) were categorized as xanthone derivatives by HPLC-DAD-MS/MS analysis but were not fully identified.

Among the twenty-five identified xanthones, seven (**X6**, **X7**, **X12**, **X14**, **X15**, **X18**, and **X28**) were upregulated in HR2 compared to HR1 cultures. Moreover, five xanthones (**X8**, **X10**, **X20**, **X21**, and **X30**) were synthesized only in HR2 cultures. Recent studies showed that *Hypericum in vitro* cultures have the potential to accumulate xanthones and their production can be manipulated by the hormonal supplementation [28], or/and by the culture type [33]. It is probable that phytohormones either facilitate or hamper the expression and activity of specific xanthone enzymes that influence xanthone accumulation in *H. perforatum* callus [33], cells [28], and root cultures [12]. Namely, Tocci et al. [12] suggested that root cultures grow continuously on nutrient media supplemented with auxins, but sometimes repetitive subcultures may induce loss of morphogenetic potential, resulting in poor or negligible secondary metabolite production. On the other hand, our results showed that *H. perforatum* HR cultures successfully grow on hormone-free media and represent a continuous source for high-level xanthone production.

Taken together, results in our study showed distinct phenolic profile between dark-grown (HR1) and photoperiod-exposed (HR2) cultures. Namely, phenolic compounds identified in HR2 cultures compared to HR1 could be distinguished in four groups: (i) compounds whose quantity increased (**F1**, **F15**, **X6**, **X7**, **X12**, **X14**,** X15**, **X18**, and **X28**), (ii) compounds whose quantity decreased (**F2**, **F4**, **F6–F10**, **F13**, **X1**, **X4**, **X5**, **X17**, **X23**, and **X32**), (iii) compounds that were not detectable (**X11**, **X13**, **X16**, **X19**, **X22**, **X25**, and **X26**), and (iv) compounds that were *de novo* synthesized (**F3**, **F5**, **F11**, **F12**, **F14**, **X8**, **X10**, **X20**, **X21**, and **X30**). Consequently, results from our experiments demonstrated that the exposure of HR2 cultures to photoperiod leads to alternations in their biosynthetic potentials.

Recent study showed that the phenolic biosynthesis and flavonoids formation are light-dependent processes [41]. Moreover, changes in light intensity are capable of inducing the production of flavonoids and total phenolics in plants [42]. Therefore, *de novo* biosynthesis and accumulation of phenolic acids, flavonols, and xanthones in HR2 cultures are not surprising since considerable evidence now shows that many of the enzymes in the phenylpropanoid/flavonoid pathway could be upregulated by light. In addition, Abbasi et al. [43] demonstrated light-stimulated accumulation of phenolic acids and phenylalanine ammonia lyase (PAL) activity in *Echinacea purpurea* HR cultures. Considering results from our study, we could hypothesize that shifting the dark-grown HR to photoperiod might induce a short-term “light-stress” response. In this view, the presence of light could induce a variety of responses along with metabolic changes that directly or indirectly trigger a “later” increase in xanthone accumulation. On the other hand, our results showed that photoperiod has an inhibitory effect on the accumulation of flavan-3-ols in HR2 cultures. Possible reasons for downregulation of flavan-3-ols could be due to the activation of their catabolism and/or reaction to unidentified products that exist in photoperiod-exposed cultures. Therefore, photoregulation of phenolic compounds biosynthesis in *H. perforatum* HR may offer additional advantages of quantitative and qualitative improvements of these medicinally important metabolites.

## 4. Conclusions

In conclusion, *H. perforatum* HR cultures provided a promising system for the production of various groups of phenolic compounds. Distinct phenolic profile between dark-grown and photoperiod-exposed HR cultures was shown as detailed for the first time. HR cultures grown under photoperiod can be proposed as a useful source for accumulation of phenolic acids and flavonols, while dark-adapted HR represent an alternative tool for flavan-3-ol production. More importantly, both HR cultures synthesized and stored significant quantities of xanthones. The use of the results reported here might contribute to further study on photoregulation and optimal control of secondary metabolite production in *H. perforatum* HR cultures.

## Figures and Tables

**Table 1:** Retention times, UV, and mass spectral data of phenolic acids, flavonols, and flavan-3-ols in *Hypericum perforatum* dark-grown (HR1) and photoperiod-exposed (HR2) hairy root culture extracts^a^.

Peak no.	Compounds	*t*_*R*_ (min)	UV (nm)	[M–H]^–^ (*m*/*z*)	–MS^2^ [M–H]^–^ (*m*/*z*)	HR1 (mg·100 g^−1^ DW ± S.D.)	HR2 (mg·100 g^−1^ DW ± S.D.)
Phenolic acids							
**F1**	Quinic acid	3.9	262, 310	191	173, **127**	166.77 ± 1.20	233.14 ± 19.31
**F3**	3-*p*-Coumaroylquinic acid	19.9	314	337	191, **163**	n.d.	12.18 ± 0.92
**F5**	3-Feruloylquinic acid	25.3	314	367	**193**	n.d.	5.87 ± 0.26

Flavonols							
**F8**	Quercetin 6-*C*-glucoside	33.9	256, 356	421	331, **301**	2.99 ± 0.79	1.74 ± 0.11
**F10**	Isorhamnetin *O*-hexoside	38.1	254, 356	477	**316**, 315, 271	11.80 ± 0.94	1.74 ± 0.09
**F11**	Kaempferol hexoside	41.2	256, 266, 350	447	**285**	n.d.	1.99 ± 0.26
**F12**	Hyperoside (quercetin 3-*O*-galactoside)	43.8	264, 296 sh, 354	463	**301**	n.d.	2.77 ± 0.18
**F13**	Rutin (quercetin 3-*O*-rutinoside)	44.9	263, 298 sh, 356	609	**301**	14.72 ± 2.16	5.46 ± 0.43
**F14**	Quercetin acetylglycoside	48.1	254, 298, 358	505	463, 445,** 301**	n.d.	3.94 ± 0.10
**F15**	Kaempferol	59.5	256, 266, 350	285	/	3.92 ± 0.38	6.26 ± 0.37

Flavan-3-ols							
**F2**	Catechin	19.5	280	289	**245**, 205	27.28 ± 3.20	2.62 ± 0.08
**F6**	(Epi)catechin	29.9	280	289	**245**, 205	184.85 ± 12.92	133.36 ± 15.19
**F4**	Proanthocyanidin dimer	24.5	280	577	559, 451, **425**, 407, 289	146.95 ± 9.13	56.61 ± 2.65
**F7**	Proanthocyanidin dimer	33.4	280	577	559, 451, **425**, 407, 289	41.43 ± 1.03	0.76 ± 0.08
**F9**	Proanthocyanidin dimer	36.8	280	577	559, 451, **425**, 407, 289	29.24 ± 2.47	24.93 ± 0.15

^a^n.d.: not detected; DW: dry weight; sh: shoulder; *t*_*R*_: retention time. MS^2^ ions in bold indicate the base peak. For information on peak numbers, see Figure 1.

**Table 2:** Retention times, UV, and mass spectral data of xanthones in *Hypericum perforatum* dark-grown (HR1) and photoperiod-exposed (HR2) hairy root culture extracts^a^.

Peak no.	Compounds	*t*_*R*_ (min)	UV (nm)	[M–H]^–^ (*m*/*z*)	–MS^2^ [M–H]^–^ (*m*/*z*)	HR1 (mg·100 g^−1^ DW ± S.D.)	HR2 (mg·100 g^−1^ DW ± S.D.)
**X1**	Mangiferin	37.3	238, 256, 312, 362	421	331, 301, **258**	1383.25 ± 88.91	669.67 ± 24.12
**X2**	Xanthone derivative 1	45.8	208, 257, 322, 374	441	423, 397, 373, 305, 257, 229	109.47 ± 9.81	n.d.
**X3**	Xanthone derivative 2	46.2	242, 306	367	**287**	635.06 ± 18.52	600.59 ± 39.62
**X4**	1,3,5,6-Tetrahydroxyxanthone dimer	50.2	252, 284, 328	517	499, 468, 446, 391, **365**	821.61 ± 28.39	692.94 ± 19.28
**X5**	1,3,6,7-Tetrahydroxyxanthone dimer	53.9	238, 254, 312, 364	517	517, 469, 447, 379, **257**	522.56 ± 25.44	88.31 ± 2.88
**X6**	1,3,5,6-Tetrahydroxyxanthone	55.4	250, 282, 328	259	**229**, 213, 187	190.17 ± 20.73	949.35 ± 51.71
**X7**	1,3,6,7-Tetrahydroxyxanthone	55.8	236, 254, 314, 364	259	231, **215**, 187, 147	167.14 ± 9.52	874.85 ± 31.24
**X8**	Tetrahydroxy-one-methoxyxanthone	57.7	254, 286, 328	289	**274**, 175	n.d.	448.65 ± 9.44
**X9**	Xanthone derivative 3	59.2	244, 280, 316	353	**273**	n.d.	276.57 ± 9.29
**X10**	1,3,5-Trihydroxy-6-methoxyxanthone	63.3	250, 284, 326	273	**258**,225	n.d.	150.86 ± 12.62
**X11**	Mangiferin *C*-prenyl isomer	73.5	238, 260, 312, 372	489	399, 369, **327**	433.68 ± 82.56	n.d.
**X12**	1,3,6,7-Tetrahydroxyxanthone 8-prenylxanthone	73.9	248, 312, 366	327	325, **297**, 258, 201	547.65 ± 15.21	737.48 ± 65.39
**X13**	1,3,5,6-Tetrahydroxyxanthone 8-prenylxanthone	74.9	242, 260, 320, 368	327	325, **297**, 258, 201	368.17 ± 21.70	n.d.
**X14**	1,3,7-Trihydroxy-2-(2-hydroxy-3-methyl-3-butenyl)xanthone	75.3	238, 260, 314, 388	327	**309**, 257	588.66 ± 49.31	854.53 ± 31.88
**X15**	Toxyloxanthone	76.2	242, 262, 330, 384	325	307, 283, 272	577.03 ± 5.09	1542.09 ± 129.21
**X16**	1,3,7-Trihydroxy-6-methoxy-8-prenylxanthone	76.5	240, 260, 318, 370	341	326, 311, **297**, 285	650.13 ± 34.77	n.d.
**X17**	1,3,6,7-Tetrahydroxyxanthone 2-prenylxanthone	76.7	248, 312, 368	327	325, 283, **271**	1402.03 ± 85.98	656.33 ± 37.25
**X18**	*γ*-Mangostin isomer	77.1	254, 286, 324	395	326, 283, **271**	1226.31 ± 185.52	1480.32 ± 130.06
**X19**	1,3,6-Trihydroxy-7-methoxy-8-prenylxanthone	77.2	240, 256, 312, 370	341	293, **256**	3240.28 ± 140.14	n.d.
**X20**	1,3,5,6-Tetrahydroxyxanthone 2-prenylxanthone	77.4	238, 260, 318, 372	327	297, **258**	n.d.	699.36 ± 49.61
**X21**	Paxanthone	78.0	244, 264, 324, 386	339	**324**, 307	n.d.	4040.70 ± 209.82
**X22**	*γ*-Mangostin isomer	78.9	260, 316, 370	395	351, **339**, 326, 283	3629.15 ± 338.08	n.d.
**X23**	Trihydroxy-1-methoxy-*C*-prenylxanthone	79.4	260, 286, 314	341	**326**	11314.34 ± 469.01	10067.14 ± 561.72
**X24**	Xanthone derivative 4	79.9	260, 308, 374	295	**277**, 251, 195, 171	n.d.	2778.02 ± 81.11
**X25**	*γ*-Mangostin	80.0	246, 262, 320	395	351, 339, 326, **283**	7861.71 ± 415.11	n.d.
**X26**	Banaxanthone D	80.2	244, 268, 332	461	393, 341, **297**	1784.69 ± 88.90	n.d.
**X27**	Xanthone derivative 5	80.5	254, 310	355	340, 325, **297**, 285, 271	2266.19 ± 191.89	1765.42 ± 36.19
**X28**	Garcinone E	81.2	256, 286, 332	463	394, 351, **339**, 297, 285	8229.95 ± 537.14	10844.13 ± 288.29
**X29**	Xanthone derivative 6	82.2	262, 288, 322	393	/	421.44 ± 36.66	370.43 ± 45.16
**X30**	Banaxanthone E	82.6	252, 302, 330	477	419, 393, **339**, 297	n.d.	499.91 ± 38.44
**X31**	Xanthone derivative 7	83.6	270, 330, 400	467	398, 383, 327, 271, 234	n.d.	429.57 ± 7.82
**X32**	Garcinone C	83.9	286, 340	413	369, 344, **301**, 233	1185.94 ± 149.05	943.63 ± 55.98
**X33**	Xanthone derivative 8	84.4	254, 284, 326	481	**412**, 397, 327, 271, 234	562 ± 38.99	126.80 ± 1.69

^a^n.d.: not detected; DW: dry weight; sh: shoulder; *t*_*R*_: retention time. MS^2^ ions in bold indicate the base peak. For information on peak numbers, see Figure 1.

## Abbreviations

DAD: Diode array detection
ESI-MS: Electrospray ionisation-mass spectrometry
HPLC: High-performance liquid chromatography
HR: Hairy roots
LC-MS: Liquid chromatography-mass spectrometry
[M–H]^−^: Negative molecular ion
MS^*n*^: Collision fragment ions
PCR: Polymerase chain reaction.
